# A nomogram for one-year risk of death after hip fracture

**DOI:** 10.3389/fmed.2025.1500049

**Published:** 2025-05-30

**Authors:** Jiale Guo, Liuyang Shi, Kehai Shi, Ru Dai, Jian Wang, Yehai Li

**Affiliations:** Department of Orthopedics, Chaohu Hospital of Anhui Medical University, Hefei, China

**Keywords:** nomograms, hip fractures, mortality, surgical procedures, conservative treatment

## Abstract

**Background:**

Hip fractures are catastrophic events with a significant risk of mortality, making early identification of high-risk patients crucial. While previous studies have primarily focused on post-surgical mortality in hip fracture patients, less attention has been given to those who did not undergo surgery. This study aimed to develop a nomogram to predict 1-year mortality in older adults following hip fractures.

**Methods:**

Patients hospitalized with hip fractures at a university hospital between May 2016 and December 2021 were included. Participants were randomly divided into training and validation cohorts (70:30 ratio). After selecting key variables, the nomogram was constructed, and its performance was evaluated in both cohorts.

**Results:**

A total of 619 patients were included, with 136 (21.97%) experiencing mortality within one year. LASSO regression was used to account for multicollinearity, selecting variables such as age, coronary heart disease, surgery, hemoglobin, aspartate transaminase, and blood urea nitrogen. The nomogram achieved AUCs of 0.83 (95% CI: 0.78–0.88) and 0.81 (95% CI: 0.73–0.89) in the training and validation cohorts, respectively, demonstrating excellent calibration and clinical utility.

**Conclusion:**

The nomogram effectively predict 1-year mortality risk in older adults following hip fractures.

## 1 Introduction

Hip fractures in older adults are a devastating event with a high risk of mortality, accounting for approximately 5% of all-cause deaths worldwide due to their severe health impacts (1). s one of the most common and serious osteoporotic fractures, hip fractures occur primarily due to weakened bone strength caused by osteoporosis, making them a critical public health issue, particularly in aging populations. Osteoporotic fractures like hip fractures not only endanger lives but also impose a significant financial burden on global healthcare systems (2). The 1-year mortality rate after hip fracture typically surpasses 20%, even in high-income countries (3). Additionally, hip fractures are expensive to treat, costing up to $14,000 per patient in the United States (3). It is estimated that 14.2 million hip fractures will occur worldwide in 2019, with an age-standardized incidence of 182 per 100,000 people (4). Meanwhile, due to the aging of the global population, the absolute number of hip fracture events continues to increase, despite a decline in the incidence of fractures in developed countries (5). Hip fractures, as a major form of osteoporotic fracture, have thus emerged as a growing public health crisis. And as an absolutely devastating event, death can cause severe suffering and burden to the patient’s family and society. Therefore, it is necessary to identify high-risk patients by predicting 1-year mortality after hip fracture in the elderly and to intervene early.

Previous related studies have focused on studies of mortality in patients after hip fracture surgery, and few have focused on studies of patients treated conservatively. At the same time, many studies have shown that conservative treatment of hip fractures in the elderly is associated with high mortality (6–12). In addition, we understand that there are a certain number of cases of hip fractures in elderly people who are admitted to hospitals for conservative treatment without contraindications to surgery, and this situation is particularly prominent in less economically developed regions. Therefore, if mortality at one year after hip fracture could be predicted early on admission, especially being able to predict the risk of death under two different decision-making scenarios, surgical and non-surgical treatment, separately for visual comparison could help to improve the situation to a certain extent.

Our aim in this study was to develop a simple, visual nomogram based on patients’ admission data (including general demographics, chronic diseases and routine test indicators) combined with surgery or not to predict different 1-year risks of death after hip fracture in elderly people under different decisions of whether to undergo surgery or not in order to assist physicians in making decisions in conjunction with the patient’s family.

## 2 Materials and methods

### 2.1 Data collection

In this study, patients hospitalized for hip fractures at a university hospital from May 2016 to December 2021 were collected. Relevant medical record data is extracted from the electronic medical record system, and the patient’s prognosis is followed up to determine the patient’s specific survival period. This data was accessed on December 26, 2022 for research purposes. Inclusion criteria: patients who were hospitalized for hip fracture. Exclusion criteria: 1. age < 60 years; 2. pathological fractures; 2. multiple injuries; 3. patients with comorbid cancer, acute cardiac and cerebral diseases, and those who had been bedridden for a long time before the fracture; 4. partial absence of data. Similar inclusion and exclusion criteria were used in studies related to perioperative blood transfusion and postoperative pneumonia in hip fracture patients during this period at our study center (13, 14).

Data was extracted from three primary categories: patients’ baseline characteristics, commonly reported chronic conditions in geriatric populations, and key laboratory test results obtained within 24 h of admission. Additionally, we specifically included whether the patient underwent surgery. (1) General characteristics of the patients: gender (Female), age, fracture side (Left), fracture time, and fracture type (femoral neck fracture, FNF). (2) Common chronic diseases in the elderly: hypertension (HBP), coronary heart disease (CHD), diabetes mellitus (DM), cerebral infarction (CI), and chronic bronchitis (CB). (3) Laboratory test results: white blood cell count (WBC), neutrophil ratio (N), red blood cell count (RBC), hemoglobin (HB), platelet count (PLT), glucose (GLU), alanine transaminase (ALT), aspartate transaminase (AST), total bilirubin (STB), direct bilirubin (DBIL), indirect bilirubin (IBIL), albumin (ALB), globulin (GLOB), blood urea nitrogen (BUN), creatinine (Cr), potassium ion (K^+^), sodium ion (Na^+^), calcium ion (Ca^2+^), prothrombin time (PT), international normalized ratio (INR), fibrinogen (FIB), activated partial thromboplastin time (APTT), thromboplastin time (TT), prothrombin activity (PTA). For laboratory test results, the normal range may vary from hospital to hospital depending on the type of test. Therefore, to make the model generalizable, we converted it into a dichotomous variable by its exceeding the upper limit or being below the lower limit in combination with the clinical. Data extraction and follow-up were performed independently by two researchers, and a third independent researcher verified the accuracy of the data.

The study was approved by the hospital’s ethics review committee (ethics number: KYXM-202212-017, approved on December 15, 2022). An informed consent waiver was obtained because the study was retrospective and we withheld patients’ personal information in the analysis. All procedures complied with the 1964 Declaration of Helsinki and its subsequent revisions.

### 2.2 Statistical analysis

The mean standard deviation is used to represent continuous variables that follow a normal distribution, the median (interquartile range) is used to represent continuous variables that do not follow a normal distribution, and percentages are used to represent categorical variables. The univariate analysis of the data between the two groups was done with the “CBCgrps” package. Besides, we also analyzed the difference between the different types of hip fractures in order to compare the differences between the two groups of intertrochanteric fractures and femoral neck fractures.

All of the analysis’s patients were randomized 70:30 into the training set (70%) and validation set (30%) groups. To identify the key variables associated with mortality in elderly people one year after hip fracture, we first performed correlation tests for all variables. The correlation coefficient takes values in [−1, 1], the larger its absolute value, the stronger the correlation, greater than 0.4 indicates that there is a significant correlation, and the results are plotted as a hotspot plot. If there is significant multicollinearity between the variables, we will use the least absolute shrinkage and selection operator (LASSO) (15, 16) technique to screen the variables; otherwise, univariate logistic regression will be followed by multifactor logistic regression analysis. A linear model was fitted to the screened variables and backward stepwise regression was used to analyze the extent to which all the included variables contributed to the model. *P* < 0.05 was defined as significant, the variables with insignificant contributions were excluded, and the remaining ones were used as the final incorporated variables to construct the model. And based on the results, a nomogram was drawn and a dynamic web calculator was constructed. The receiver operating characteristic (ROC) (17, 18) curves were used to assess the predictive performance of the nomogram. The higher the area under the curve (AUC) of the ROC, the better the model’s performance. Sampling was performed 1,000 times using Bootstrap to assess the stability and generalization performance of the models. The impact of each variable on the overall model performance was assessed by removing each variable one by one for sensitivity analysis, constructing sub-models and comparing the AUC of each model. The calibration curve was used to indicate the agreement between the predicted and actual probabilities of the analyzed model (19, 20), and the Hosmer–Lemeshow test was used to evaluate the nomogram’s goodness of fit (21). In addition, decision curve analysis (DCA) and clinical impact curves (CIC) were used to assess the clinical utility of the nomogram in decision making (22). R (version 4.1.3) software was used to implement all statistical analyses, model development, and validation in this study.

## 3 Results

### 3.1 Baseline characteristics

**FIGURE 1:**
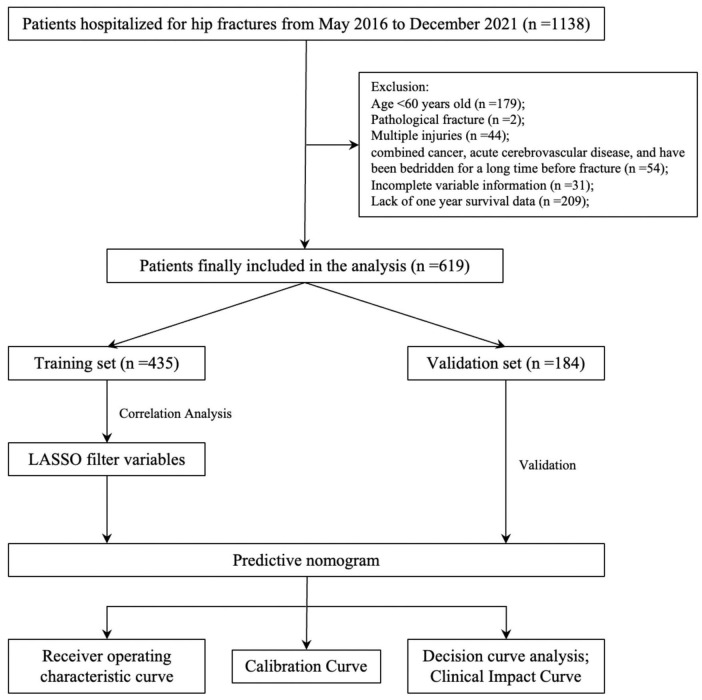
Flowchart of data screening and analysis.

Based on the inclusion and exclusion criteria, 619 patients were ultimately included in the study. The screening and entire analysis process are shown in Figure 1. There were 318 femoral neck fractures and 301 intertrochanteric fractures in the included patients, of which 10.7% (*n* = 34) and 21.3% (*n* = 64) were treated conservatively. 136 (21.97%) patients died within 1 year of the fracture. Table 1 summarizes the characteristics of all enrolled patients and divides them into two groups to examine the differences according to whether they died one year after the fracture. Compared to the survival group, the death group exhibited significant differences in several variables. For instance, the proportion of females was 54% in the death group versus 66% in the survival group; the median age was 84 years in the death group compared to 78 years in the survival group; the time from fracture to hospital admission was longer in the death group (*P* = 0.043); 38% of patients in the death group had CHD versus 15% in the survival group; 35% had CI compared to 24% in the survival group; 20% of patients in the death group had B versus 9% in the survival group; 43% of patients in the death group sustained femoral neck fractures compared to 54% in the survival group; and only 57% of the death group underwent surgical treatment, in contrast to 97% in the survival group. Furthermore, the differences between intertrochanteric fractures and femoral neck fractures are presented in Supplementary Table 1. All patients were randomized into the training set (*n* = 435, 70%) and the validation set (*n* = 184, 30%), and baseline information for both groups is shown in Table 2.

**TABLE 1:** Characteristics of all enrolled patients, the alive group, and the death group.

Variables	Total (*n* = 619)	Alive (*n* = 483)	Death (*n* = 136)	*P*
Female, *n* (%)	392 (63)	318 (66)	74 (54)	0.019*
Age, median (Q1, Q3)	79 (72, 85)	78 (71, 84)	84 (78.75, 87.25)	< 0.001*
Left, *n* (%)	299 (48)	235 (49)	64 (47)	0.817
Fracture time [days], median (Q1, Q3)	1 (1, 2)	1 (1, 2)	1 (1, 3)	0.043*
HBP, *n* (%)	320 (52)	248 (51)	72 (53)	0.817
CHD, *n* (%)	126 (20)	74 (15)	52 (38)	< 0.001*
DM, *n* (%)	118 (19)	84 (17)	34 (25)	0.061
CI, *n* (%)	164 (26)	116 (24)	48 (35)	0.012*
CB, *n* (%)	69 (11)	42 (9)	27 (20)	< 0.001*
FNF, *n* (%)	318 (51)	260 (54)	58 (43)	0.027*
Surgery, *n* (%)	521 (84)	444 (92)	77 (57)	< 0.001*
WBC [> 10 × 10^9^/L], *n* (%)	116 (19)	82 (17)	34 (25)	0.046*
N [> 70%], *n* (%)	496 (80)	388 (80)	108 (79)	0.908
RBC [< lower limitation], *n* (%)	349 (56)	246 (51)	103 (76)	< 0.001*
HB [< lower limitation], *n* (%)	388 (63)	279 (58)	109 (80)	< 0.001*
PLT [< 100 × 10^9^/L], *n* (%)	93 (15)	71 (15)	22 (16)	0.772
GLU [> 6.1 mmol/L], *n* (%)	296 (48)	221 (46)	75 (55)	0.066
ALT [> 40 u/L], *n* (%)	36 (6)	20 (4)	16 (12)	0.002*
AST [> 40 u/L], *n* (%)	43 (7)	23 (5)	20 (15)	< 0.001*
STB [> 17.1 umol/L], *n* (%)	301 (49)	243 (50)	58 (43)	0.138
DBIL [> 6.8 umol/L], *n* (%)	281 (45)	219 (45)	62 (46)	1
IBIL [> 10.2 umol/L], *n* (%)	333 (54)	276 (57)	57 (42)	0.002*
ALB [< 35 g/L], *n* (%)	201 (32)	131 (27)	70 (51)	< 0.001*
GLOB [> 35 g/L], *n* (%)	51 (8)	38 (8)	13 (10)	0.648
BUN [> 9.5 mmol/L], *n* (%)	152 (25)	84 (17)	68 (50)	< 0.001*
Cr [> 97 umol/L], *n* (%)	123 (20)	72 (15)	51 (38)	< 0.001*
Ka^+^ [< 3.5 mmol/L], *n* (%)	157 (25)	127 (26)	30 (22)	0.373
Na^+^ [< 135 mmol/L], *n* (%)	36 (6)	22 (5)	14 (10)	0.02*
Ca^2+^ [< 2.25 mmol/L], *n* (%)	533 (86)	412 (85)	121 (89)	0.341
PT [> 13 s], *n* (%)	401 (65)	297 (61)	104 (76)	0.002*
INR > 1.15, *n* (%)	71 (11)	50 (10)	21 (15)	0.135
FIB [> 4 g/L], *n* (%)	262 (42)	192 (40)	70 (51)	0.019*
APTT [> 31.3 s], *n* (%)	586 (95)	457 (95)	129 (95)	1
TT [> 21 s], *n* (%)	13 (2)	7 (1)	6 (4)	0.044*
PTA [< 70%], *n* (%)	305 (49)	227 (47)	78 (57)	0.042*

*P < 0.05. For RBC, the lower limitation is 4 × 10^12^/L for males and 3.5 × 10^12^/L for females. For HB, the lower limitation is 120 g/L for males and 110 g/L for females.

**TABLE 2:** Characteristics of the training and validation sets.

Variables	dev (*n* = 435)	vad (*n* = 184)	*p*
Female, *n* (%)	280 (64)	112 (61)	0.463
Age, median (Q1, Q3)	80 (72.5, 85)	77 (71.75, 84)	0.054
Death, *n* (%)	97 (22)	39 (21)	0.844
Left, *n* (%)	208 (48)	91 (49)	0.775
Fracture time [days], median (Q1, Q3)	1 (1, 2)	1 (1, 3)	0.044*
HBP, *n* (%)	231 (53)	89 (48)	0.323
CHD, *n* (%)	92 (21)	34 (18)	0.519
DM, *n* (%)	80 (18)	38 (21)	0.587
CI, *n* (%)	120 (28)	44 (24)	0.397
CB, *n* (%)	48 (11)	21 (11)	1
FNF, *n* (%)	226 (52)	92 (50)	0.721
Surgery, *n* (%)	361 (83)	160 (87)	0.265
WBC [> 10 × 10^9^/L], *n* (%)	90 (21)	26 (14)	0.072
N [> 70%], *n* (%)	352 (81)	144 (78)	0.517
RBC [< lower limitation], *n* (%)	239 (55)	110 (60)	0.307
HB [< lower limitation], *n* (%)	267 (61)	121 (66)	0.348
PLT [< 100 × 10^9^/L], *n* (%)	67 (15)	26 (14)	0.778
GLU [> 6.1 mmol/L], *n* (%)	202 (46)	94 (51)	0.332
ALT [> 40 u/L], *n* (%)	28 (6)	8 (4)	0.408
AST [> 40 u/L], *n* (%)	35 (8)	8 (4)	0.139
STB [> 17.1 umol/L], *n* (%)	220 (51)	81 (44)	0.161
DBIL [> 6.8 umol/L], *n* (%)	206 (47)	75 (41)	0.156
IBIL [> 10.2 umol/L], *n* (%)	244 (56)	89 (48)	0.094
ALB [< 35 g/L], *n* (%)	143 (33)	58 (32)	0.815
GLOB [> 35 g/L], *n* (%)	34 (8)	17 (9)	0.668
BUN [> 9.5 mmol/L], *n* (%)	106 (24)	46 (25)	0.948
Cr [> 97 umol/L], *n* (%)	89 (20)	34 (18)	0.649
Ka^+^ [< 3.5 mmol/L], *n* (%)	118 (27)	39 (21)	0.147
Na^+^ [< 135 mmol/L], *n* (%)	24 (6)	12 (7)	0.764
Ca^+^ [< 2.25 mmol/L], *n* (%)	381 (88)	152 (83)	0.131
PT [> 13 s], *n* (%)	289 (66)	112 (61)	0.217
INR > 1.15, *n* (%)	50 (11)	21 (11)	1
FIB [> 4 g/L], *n* (%)	170 (39)	92 (50)	0.015*
APTT [> 31.3 s], *n* (%)	414 (95)	172 (93)	0.508
TT [> 21 s], *n* (%)	7 (2)	6 (3)	0.222
PTA [< 70%], *n* (%)	213 (49)	92 (50)	0.883

dev: training set. vad: validation set.

*P < 0.05.

### 3.2 Variable selection and construction of nomogram

**FIGURE 2:**
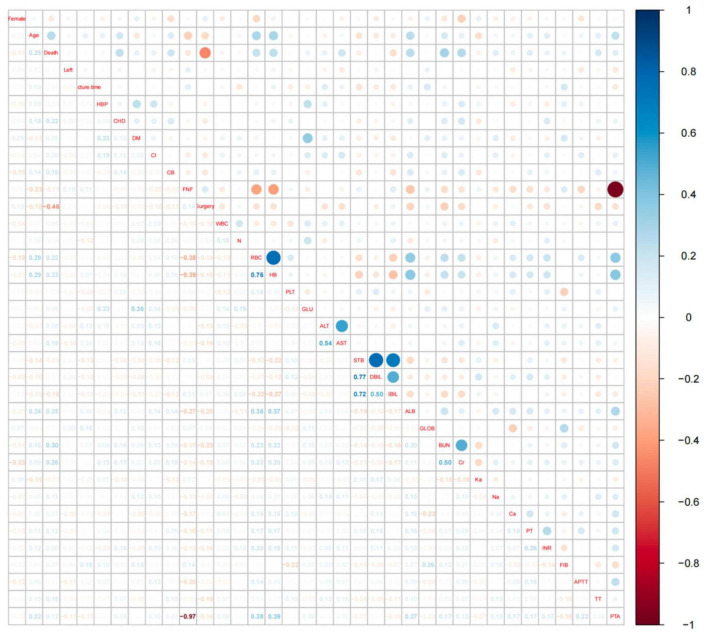
Hotspot plot for correlation analysis of all variables. The vertical coordinates on the right side of Figure 2 represent the correlation coefficients, values greater than 0 indicate a positive correlation and are shown in blue in the figure, values less than 0 indicate a negative correlation and are shown in red in the figure. The names of the variables on the diagonal indicate the rows and columns corresponding to the variable, the area above the diagonal shows the correlation between the two variables in dots, and the area below the diagonal shows the specific value of the correlation between the two variables. An absolute value of more than 0.4 for the correlation coefficient indicates a significant correlation.

**FIGURE 3:**
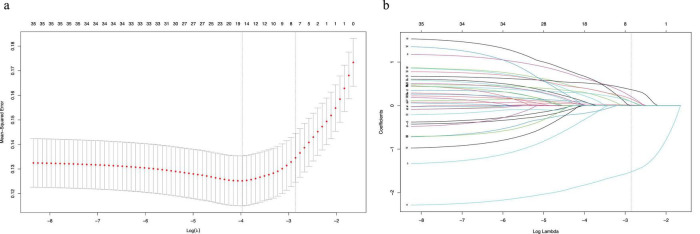
The potential risk factors were selected using the LASSO regression. **(a)** Graph of cross-validation results. The vertical line on the left side represents λ min, and the vertical line on the right side represents λ 1se. λ min refers to the λ value corresponding to the minimum mean squared error (MSE) among all λ values; λ 1se refers to the λ value corresponding to the simplest and best model obtained after cross-validation within a square difference range of λ min. **(b)** Trend graph of variance filter coefficients. Each color curve represents a trend in variance coefficient change.

**FIGURE 4:**
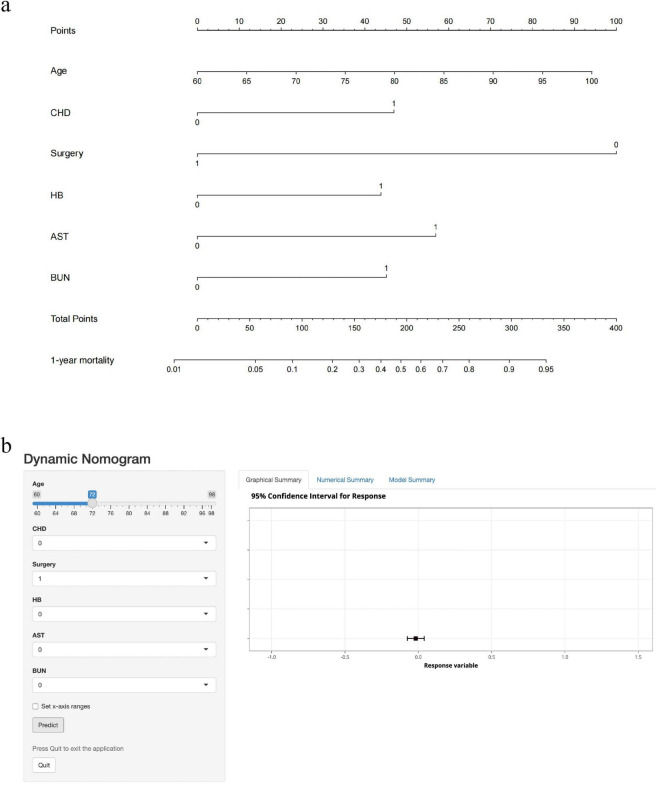
Nomogram and dynamic web calculator to predict 1-year mortality after hip fracture in older adults. CHD: 1 means the patient has CHD; 0 means none. Surgery: 1 means the patient underwent surgery; 0 means no surgery. HB: 1 means the patient is anemic; 0 means no anemia. AST, BUN: 1 means the patient’s test result exceeds the upper limit of normal value; 0 means the upper limit of normal value is not exceeded. **(a)** Nomogram to predict 1-year mortality after hip fracture in the elderly. According to the patient’s status of each item, the first row of “Points” is selected, and the cumulative score of the six rows is recorded as “Total Points,” and the corresponding “1-year mortality” value is selected to represent the 1-year risk of death after hip fracture. **(b)** Dynamic web calculator to predict 1-year mortality after hip fracture in the elderly. Click on the “Predict” button and the “Graphical Summary” on the right shows the corresponding risk values (95% confidence interval) in graphical form, the “Numerical Summary” on the right shows the specific risk values (95% confidence interval), and the “Model Summary” on the right shows the specific information of the model.

We extracted 35 variables from each patient. The results of the correlation test between variables showed significant multicollinearity among several variables (23) (Figure 2). Based on the results of the correlation test, we chose LASSO regression to perform variable screening. The results showed that when the lambda value was chosen as lambda.1se (0.05750), a total of eight variables with non-zero coefficients were screened (Figure 3). In order to further simplify the model, we build generalized linear models using the filtered variables and assess the relative contributions of each component. After excluding 2 variables with insignificant contributions, the remaining 6 variables (age, CHD, surgery, HB, AST, and BUN) were used to construct the final prediction model (Table 3) and visualized as a nomogram (Figure 4a). In addition, we designed an online web calculator to assess the 1-year risk of death after hip fracture in older adults based on the results (Figure 4b)^1^.

**TABLE 3:** Contribution of the generalized linear model.

Variables	OR [95% CI]	*p*
(Intercept)	0.01 [0.00, 0.13]	0.002
Age	1.05 [1.01, 1.09]	0.012
CHD	2.49 [1.33, 4.64]	0.004
Surgery	0.13 [0.07, 0.24]	< 0.001
HB	2.05 [1.02, 4.25]	0.048
AST	3.10 [1.25, 7.66]	0.014
ALB	1.57 [0.86, 2.85]	0.136*
BUN	2.01 [1.04, 3.88]	0.038
Cr	1.71 [0.86, 3.41]	0.125*

OR, odds ratio; CI, confidence interval; CHD, coronary heart disease; HB, hemoglobin; AST, aspartate transaminase; ALB, albumin; BUN, blood urea nitrogen; Cr, creatinine.

*P < 0.05.

### 3.3 Evaluation of nomogram

**FIGURE 5:**
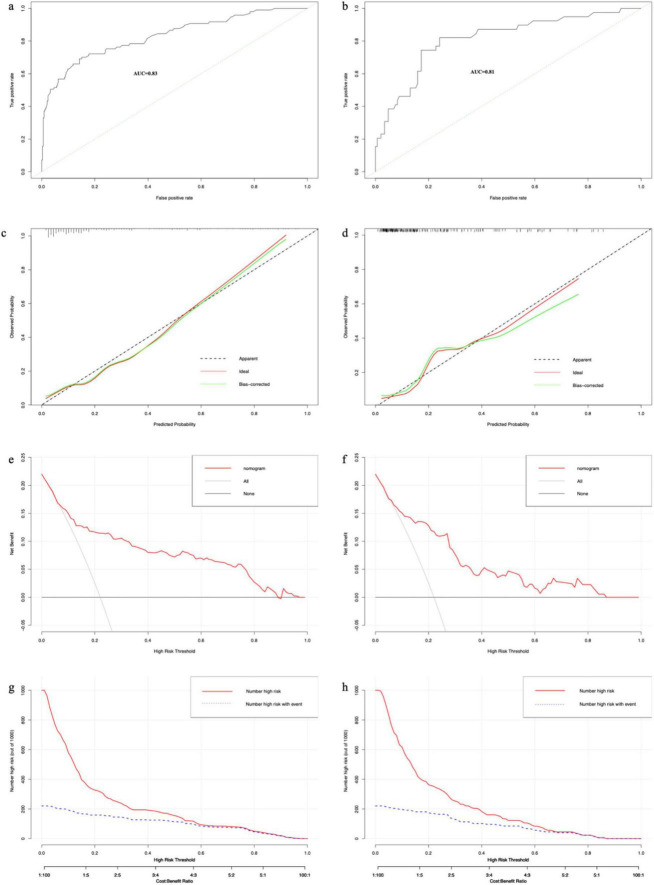
The results of each performance evaluation of the nomogram. **(a)** ROC curve in the training set. **(b)** ROC curve in the validation set. **(c)** Calibration curve in the training set. **(d)** Calibration curve in the validation set. **(e)** DCA curve in the training set. **(f)** DCA curve in the validation set. **(g)** CIC in the training set. **(h)** CIC in the validation set.

We plotted ROC curves based on the nomogram model. The AUC values of the nomogram model were 0.83 (95% CI: 0.78–0.88) in the training set (Figure 5a) and 0.81 (95% CI: 0.73–0.89) in the validation set (Figure 5b), respectively. Similarly, the results from 1000 bootstrap resamples indicate that the nomogram exhibits good stability and generalizability, as detailed in Supplementary Table 2. After sequentially excluding Age, CHD, Surgery, HB, AST, and BUN, the model’s AUC values were 0.82, 0.83, 0.79, 0.82, 0.82, and 0.82, respectively. It shows that the model has strong discriminative ability in both the training and validation sets. The calibration curves of this prediction model show good agreement between predictions and observations in the training set (Figure 5c) and the validation set (Figure 5d), with the Hosmer–Lemeshow test showing non-significant *p*-values of 0.55 and 0.12, respectively. The DCA curves show that for both the training set (Figure 5e) and the validation set (Figure 5f) if the threshold probability is in the range of 0.1 to 0.9, the prediction model achieves a higher net return than the “all intervention” or “no intervention” strategy. The CIC results showed that the number of high-risk patients (number of deaths predicted using the nomogram) and the number of incident high-risk patients (number of true-positive deaths) were highly matched, with threshold probabilities above 0.62 in the training set (Figure 5g) and 0.65 in the validation set (Figure 5h), respectively.

## 4 Discussion

In this study, we developed and validated a nomogram for predicting mortality in older adults one year after a hip fracture. The nomogram includes six variables: age, CHD, surgery, HB, AST, and BUN. The results of the nomogram assessment showed good discriminatory efficacy, calibration efficacy, and clinical utility with good clinical utility. The results of the nomogram assessment showed good discriminatory efficacy, calibration efficacy, and clinical utility. Due to the large number of variables included, we conducted a prior correlation analysis among the variables. Based on the results of the analysis, LASSO regression was used to screen the variables and avoid interactions between the variables that were eventually included in the model. In addition, our data in this study except for whether surgery was performed were collected within 24 h of admission, which to some extent avoids bias due to treatment effects at different levels of hospitals, making the column line graphs a good reference at different levels of hospitals.

This is the first study to use the nomogram to predict 1-year mortality after hip fracture in older adults. Although there are many previous studies similar to ours, they were mainly used to predict the risk of death after hip fracture surgery in older adults (24–29). Only Cary et al. (30) and Kitcharanant et al. (31) shared our concern for this subset of patients who had hip fractures but did not receive surgical treatment. However, they are all predictive models built based on machine learning algorithms. This algorithm, despite the possibility of better model performance, is prone to overfitting and is difficult to interpret for a single individual (32–34). In contrast, the nomogram is easy to use as a simple graphical representation of the results of the statistical algorithm (35), allowing the inclusion of relevant determinants of disease in the prognosis (36–39). This study included 619 elderly patients with hip fractures, with an overall mortality rate of 22% (*n* = 136), similar to that reported in previous studies. Results from the National Hospital Records and Official Death Records of England 2015–2017 showed a 1-year mortality rate of 27.2% for patients with hip fractures (*n* = 169,646) (40). An analysis of relevant trials published between 1981 and 2012 showed that the 1-year mortality rate after hip fracture remained stable over time at about 20% (41). In Spain, the cumulative 1-year mortality rate after fracture-sparing hip fracture was 33% in 1999–2015 (42). However, the mortality rate 12 months after fracturing in the study of Kitcharanant et al. was only 12.6% (31). This difference may be due to the different percentages of conservative treatment in the sample size: 16% of conservative treatment in our study compared to only 6.5% in the study by Kitcharanant et al. (31).

Many studies are showing that conservative treatment is associated with a high risk of death. In the Singapore region, patients treated conservatively had a four-fold higher mortality rate one year after fracture and a three-fold higher mortality rate after two years compared to the surgical group (43). A survey of population-based registry data from the New York State Planning and Research Collaborative System (SPARCS) showed a 30-day post-discharge mortality rate of 4.5% for surgically treated patients and 10.7% for non-surgically treated patients (44). Results from a matched cohort study also showed that the mean life expectancy after hip fracture was significantly shorter in the non-operative group than in the operative group (221 versus 1,024 days; *P* < 0.0001), and 1-year mortality was significantly higher than in the matched operative cohort (45). Poor prognosis for elderly patients who refuse surgery after hip fracture due to financial burden and medical problems (9). By analyzing data from the US. Medicare population from 1991 to 2008, patients with conservative treatment of hip fractures accounted for about 5% of all admissions to the hospital (46). In Canada, non-surgical treatment declined from 8.3% in 1990–1994 to 5.1% in 2010–2014 (47). A significant number of patients still choose conservative treatment after hip fracture, and this trend is even greater in less economically developed regions (48). Therefore, the inclusion of surgery or not in the nomogram as an independent prognostic factor significantly associated with mortality (49) is of great importance. This is the first study to include surgery or not as a key variable in the nomogram used to predict the risk of death from hip fractures in older adults. This nomogram allows for predicting the different risks of death for patients with different decisions of whether to operate or not. And it provides visual evidence support to enhance the implementation of shared decision making (50). Furthermore, for patients identified as high risk, more proactive early intervention measures may be implemented. For example, high-risk patients receiving conservative treatment should be provided with more aggressive nursing care and rehabilitation recommendations. However, there is still a lack of evidence regarding which specific interventions can most effectively improve subsequent mortality and quality of life in conservatively managed patients (51). Although this subgroup represents a relatively small proportion of the overall population, future prospective studies should focus on their survival, rehabilitation, and mental health, as well as on the development of cost-effective intervention strategies to improve prognosis.

However, our study has some limitations. First, it was a retrospective study, so there may be some degree of selection and analysis bias, and further prospective studies are needed. Second, although our sample size was able to meet the minimum requirements for constructing the model (the number of positive samples in the training set was at least ten times the number of predictor variables (52–55)), the sample size included in the study was still not large enough, which may lead to a lack of robustness of the conclusions. Third, this study did not separately analyze intertrochanteric fractures and femoral neck fractures. Future research with larger sample sizes is needed to independently evaluate the impact of conservative treatment on mortality risk in each of these fracture subtypes. Finally, the prediction model we constructed, like the vast majority of studies, uses data from a single center and should be treated with caution when applied to other regions and populations. Further validation of the performance evaluation of our model is needed with external data.

## 5 Conclusion

We found that age, CHD, surgery, HB, AST, and BUN were significant independent predictors of 1-year mortality after hip fracture in older adults and constructed a nomogram based on these variables. The model performed well, but further prospective studies and external data validation are needed.

## Data Availability

The data analyzed in this study is subject to the following licenses/restrictions: The data that support the findings of this study are available from the corresponding author upon reasonable request. Requests to access these datasets should be directed to YL, ahyylyh@163.com.

## Footnotes

1
https://jialeguo.shinyapps.io/dynamic-nomogram/
