# Supplementary material for: A nomogram for one-year risk of death after hip fracture
Source: Front Med (Lausanne). 2025 May 30;12:1500049. doi: 10.3389/fmed.2025.1500049 (PMC12162923; doi:10.3389/fmed.2025.1500049)
Supplement: Supplementary file 1 [file Table_1.docx]

**S1.** Characteristics of all enrolled patients, the ITF group, and the FNF group.

| **Variables** | **Total (n = 619)** | **ITF (n = 301)** | **FNF (n = 318)** | **p** |
| --- | --- | --- | --- | --- |
| Death, n (%) | 136 (22) | 78 (26) | 58 (18) | 0.027* |
| Female, n (%) | 392 (63) | 184 (61) | 208 (65) | 0.307 |
| Age, Median (Q1, Q3) | 79 (72, 85) | 82 (74, 86) | 77 (71, 82) | < 0.001* |
| Left, n (%) | 299 (48) | 127 (42) | 172 (54) | 0.004* |
| Fracture time [days], Median (Q1, Q3) | 1 (1, 2) | 1 (1, 2) | 1 (1, 3) | 0.003* |
| HBP, n (%) | 320 (52) | 162 (54) | 158 (50) | 0.343 |
| CHD, n (%) | 126 (20) | 66 (22) | 60 (19) | 0.398 |
| DM, n (%) | 118 (19) | 55 (18) | 63 (20) | 0.7 |
| CI, n (%) | 164 (26) | 87 (29) | 77 (24) | 0.219 |
| CB, n (%) | 69 (11) | 44 (15) | 25 (8) | 0.011* |
| Surgery, n (%) | 521 (84) | 237 (79) | 284 (89) | < 0.001* |
| WBC [>10×10^9^/L], n (%) | 116 (19) | 69 (23) | 47 (15) | 0.013* |
| N [>70%], n (%) | 496 (80) | 245 (81) | 251 (79) | 0.505 |
| RBC [< lower limitation], n (%) | 349 (56) | 231 (77) | 118 (37) | < 0.001* |
| HB [< lower limitation], n (%) | 388 (63) | 252 (84) | 136 (43) | < 0.001* |
| PLT [<100×10^9^/L], n (%) | 93 (15) | 43 (14) | 50 (16) | 0.698 |
| GLU [>6.1mmol/L], n (%) | 296 (48) | 155 (51) | 141 (44) | 0.089 |
| ALT [>40u/L], n (%) | 36 (6) | 19 (6) | 17 (5) | 0.733 |
| AST [>40u/L], n (%) | 43 (7) | 23 (8) | 20 (6) | 0.615 |
| STB [>17.1umol/L], n (%) | 301 (49) | 136 (45) | 165 (52) | 0.112 |
| DBIL [>6.8umol/L], n (%) | 281 (45) | 139 (46) | 142 (45) | 0.764 |
| IBIL [>10.2umol/L], n (%) | 333 (54) | 146 (49) | 187 (59) | 0.013* |
| ALB [<35g/L], n (%) | 201 (32) | 139 (46) | 62 (19) | < 0.001* |
| GLOB [>35g/L], n (%) | 51 (8) | 20 (7) | 31 (10) | 0.209 |
| BUN [>9.5mmol/L], n (%) | 152 (25) | 95 (32) | 57 (18) | < 0.001* |
| Cr [>97umol/L], n (%) | 123 (20) | 76 (25) | 47 (15) | 0.002* |
| Ka^+^ [<3.5mmol/L], n (%) | 157 (25) | 65 (22) | 92 (29) | 0.045* |
| Na^+^ [<135mmol/L], n (%) | 36 (6) | 26 (9) | 10 (3) | 0.006* |
| Ca^2+^ [<2.25mmol/L], n (%) | 533 (86) | 277 (92) | 256 (81) | < 0.001* |
| PT [>13s], n (%) | 401 (65) | 218 (72) | 183 (58) | < 0.001* |
| INR>1.15, n (%) | 71 (11) | 49 (16) | 22 (7) | < 0.001* |
| FIB [>4g/L], n (%) | 262 (42) | 110 (37) | 152 (48) | 0.006* |
| APTT [>31.3s], n (%) | 586 (95) | 299 (99) | 287 (90) | < 0.001* |
| TT [>21s], n (%) | 13 (2) | 8 (3) | 5 (2) | 0.509 |
| PTA [<70%], n (%) | 305 (49) | 299 (99) | 6 (2) | < 0.001* |

ITF: Intertrochanteric Fracture

FNF: Femoral Neck Fracture

*: P <0.05
