# Supplementary material for: A nomogram for one-year risk of death after hip fracture
Source: Front Med (Lausanne). 2025 May 30;12:1500049. doi: 10.3389/fmed.2025.1500049 (PMC12162923; doi:10.3389/fmed.2025.1500049)
Supplement: Supplementary file 2 [file Table_2.docx]

**S2.** Bootstrap Validation Results of Model Performance Metrics

|  | Index.orig | Train | Test | Optimism | Index.corrected | n |
| --- | --- | --- | --- | --- | --- | --- |
| Dxy | 0.6618 | 0.6722 | 0.6525 | 0.0196 | 0.6422 | 1000 |
| Intercept | 0.0000 | 0.0000 | -0.0479 | 0.0479 | -0.0479 | 1000 |
| Slope | 1.0000 | 1.0000 | 0.9460 | 0.0540 | 0.9460 | 1000 |
| Emax | 0.0000 | 0.0000 | 0.0207 | 0.0207 | 0.0207 | 1000 |
| Brier Score | 0.1127 | 0.1101 | 0.1153 | -0.0052 | 0.1179 | 1000 |

Index.orig: Performance of the model on the original dataset (original fit results).

Training: Performance of the model on the bootstrap train dataset.

Test: Performance of the model on the bootstrap test dataset.

Optimism: The difference in performance between the training and test datasets, which reflects the degree of overfitting.

Index.corrected: The bias-corrected model performance, obtained by subtracting the optimism from the original model performance.

n: The number of bootstrap resamples (in this case, n = 1000).

Dxy: Measures the model's discrimination ability and is closely related to the C-index, C-index= $\frac{\mathrm{Dxy}+1}{2}$, the closer the Dxy is to 1, the stronger the model's classification performance.

Intercept: The calibration intercept, with an ideal value of 0, reflecting the systematic bias of the model.

Slope: The calibration slope, which reflects the model's fit accuracy; the closer it is to 1, the stronger the model's fitting precision.

Emax: The maximum calibration error; the closer it is to 0, the better the model's calibration.

Brier Score: Measures the mean squared error between the predicted probabilities and the actual outcomes; the closer it is to 0, the higher the predictive accuracy.
